# Promising Technique for Facial Nerve Reconstruction in Extended Parotidectomy

**Published:** 2015-11

**Authors:** Ithzel Maria Villarreal, Antonio Rodríguez-Valiente, Jose Ramon Castelló, Carmen Górriz, Oscar Alvarez Montero, Jose Ramon García-Berrocal

**Affiliations:** 1 *Departments of Otorhinolaryngology “Puerta de Hierro-Majadahonda” University Hospital, Madrid, Spain.*; 2 *Departments of Plastic Surgery “Puerta de Hierro-Majadahonda” University Hospital, Madrid, Spain.*

**Keywords:** Anterior Lateral Thigh flap, Facial nerve, Parotidectomy, Vascularized flap

## Abstract

**Introduction:**

Malignant tumors of the parotid gland account scarcely for 5% of all head and neck tumors. Most of these neoplasms have a high tendency for recurrence, local infiltration, perineural extension, and metastasis. Although uncommon, these malignant tumors require complex surgical treatment sometimes involving a total parotidectomy including a complete facial nerve resection. Severe functional and aesthetic facial defects are the result of a complete sacrifice or injury to isolated branches becoming an uncomfortable distress for patients and a major challenge for reconstructive surgeons.

**Case Report:**

A case of a 54-year-old, systemically healthy male patient with a 4 month complaint of pain and swelling on the right side of the face is presented. The patient reported a rapid increase in the size of the lesion over the past 2 months. Imaging tests and histopathological analysis reported an adenoid cystic carcinoma. A complete parotidectomy was carried out with an intraoperative notice of facial nerve infiltration requiring a second intervention for nerve and defect reconstruction. A free ALT flap with vascularized nerve grafts was the surgical choice. A 6 month follow-up showed partial facial movement recovery and the facial defect mended.

**Conclusion:**

It is of critical importance to restore function to patients with facial nerve injury. Vascularized nerve grafts, in many clinical and experimental studies, have shown to result in better nerve regeneration than conventional non-vascularized nerve grafts. Nevertheless, there are factors that may affect the degree, speed and regeneration rate regarding the free fasciocutaneous flap. In complex head and neck defects following a total parotidectomy, the extended free fasciocutaneous ALT (anterior-lateral thigh) flap with a vascularized nerve graft is ideally suited for the reconstruction of the injured site. Donor–site morbidity is low and additional surgical time is minimal compared with the time of a single ALT flap transfer.

## Introduction

Malignant tumors of the parotid gland account scarcely for 5% of all head and neck malignancies. Although uncommon, these malignant tumors require complex surgical treatment sometimes involving a total parotidectomy including facial nerve resection (1).

Adenoid cystic carcinoma is a locally aggressive slow-growing neoplasm with a recurrence rate and capacity for nerve dissemination (2). They account for approximately 1% of malignant tumors of the facial and oral regions and approximately 17% of the total number of salivary gland malignancies (2). 

Severe functional and aesthetic facial defects are the result of a complete sacrifice of the facial nerve or injury to isolated branches causing an uncomfortable distress for patients and a major challenge for reconstructive surgeons. 

## Case Report

A 54-year-old, healthy male patient presented with a 6 month complaint of pain and swelling on the right side of the face associated with facial paralysis. He also noted intermittent right otalgia that had worsened in the last month and a rapid increase in the size of the lesion over the past 2 months. Physical examination showed right facial paralysis (House-Brackmann IV) and pain upon palpation of the posterior region of the right parotid gland. Normal saliva secretion was observed from both Stenon ducts. 

A Magnetic Resonance Image (MRI), an Ultrasound-Guided Fine Needle Aspiration Biopsy (FNAB) and a neurophysiological examination was performed. The MRI revealed a heterogeneous enhancing soft tissue mass of 4×3×3 cm in size with irregular borders. It showed low intensity areas on T2-weighted images suggesting a malignant and infiltrative process located in the superficial and deep right parotid lobes. The FNAB showed a neoplastic proliferation of dark-staining epithelial cells with basophilic nuclei and scant cytoplasm enclosing circular cribiform pattern spaces and also solid and microcystic patterns which suggested an adenoid cystic carcinoma. The neurophysiological test indicated a peripheral neuropathy of the facial nerve predominantly of the temporo-facial branch. 

**Fig 1(A, B):**
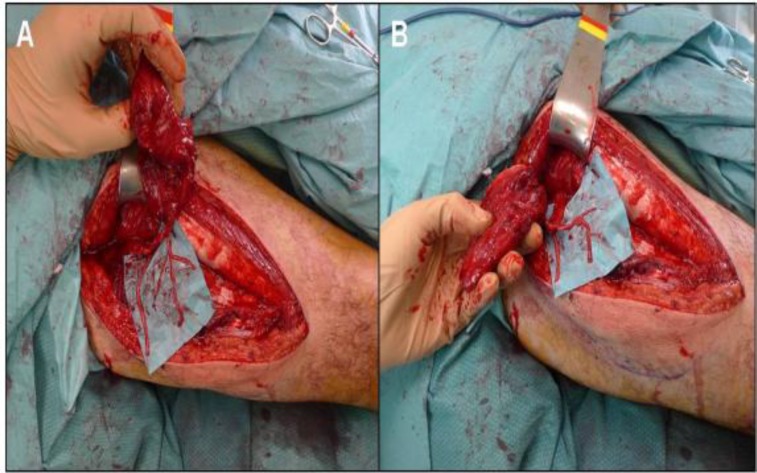
Fasciocutaneous free anterior-lateral thigh flap with donor-nerve grafts

A complete parotidectomy was carried out with an intraoperative biopsy of facial nerve infiltration until the stylomastoid foramen confirmed by histopathology results. Due to these findings and results from the first surgery a second intervention was scheduled for nerve and defect reconstruction. An open mastoidectomy was performed to remove skin from the external auditory canal. The Eustachian tube was shut down and the malleus and the incus were removed to have a better access to the Fallopian aqueduct leaving the stapes intact. The Fallopian aqueduct was carefully drilled, searching for a tumor-free facial nerve stump for an end-to-end nerve grafting. A free fasciocutaneous anterior lateral thigh (ALT) flap with a vascularized lateral femoral cutaneous nerve graft (3 distal ends and 1 proximal) was harvested and applied (Fig.1). 

**Fig 2 (C, D):**
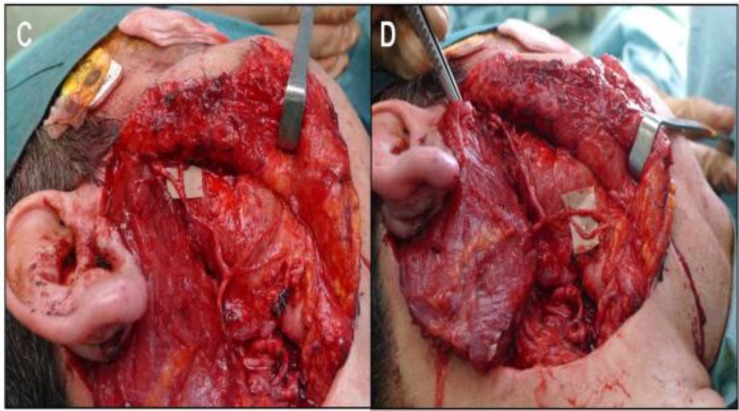
Neurorraphy of the vascularized nerve graft to the proximal facial nerve stump. The distal end of the donor nerve is dissected into fascicles allowing multiple nerve anastomoses (zygomatic and buccal branches

The vastus lateralis nerve was found to be intimately connected with the descending branch of the lateral circumflex femoral artery and its perforating vessels. It was elevated while left attached to the vascular pedicle adding consecutively the dissection of 3 to 4 distal branches and incorporating them to the flap harvest. The vascular end-to-end anastomoses were performed first and the placing of the nerve as an interpositional graft was performed right after. The selected vessels for the anastomoses were one artery, the superior thyroid artery, and two veins: the external jugular vein and a branch of the internal jugular vein. A neurorraphy of the vascularized nerve from the ALT was sutured to the proximal stump of the facial nerve and to both distal ipsilateral zygomatic and buccal branches (Fig. 2). 

The length of the graft used for reconstruction going from the proximal stump of the facial nerve to each peripheral branch of the facial nerve was approximately 6 cm. A 10 month follow-up period showed that partial facial movement was recovered reaching a House-Brackmann II and that the facial defect was mended. Partial recovery was observed in the zygomatic and buccal branches of the reconstructed facial nerve. The electromyography and neuronography studies (EMG/ENG), performed 8 months after the surgery, showed small polyphasic potentials with medium amplitude and active reinnervation characteristics, especially seen in the Orbicularis oculi muscle and with less intensity in the Orbicularis oris muscle. These potentials are suggestive of a regeneration phase in course. The donor site showed complete recovery with no complications nearby. Distant and local metastasis was assessed with negative outcomes. 

## Discussion

In complex head and neck defects following a total parotidectomy, the extended free fasciocutaneous ALT flap with a vascularized nerve graft is ideally suited for the reconstruction of the injured site. There are numerous ways to harvest the ALT flap, first described by Song et al (3). ALT flaps have a very versatile behavior as they may lift up as a myocutaneous flap, fasciocutaneous flap, chimera flap and countless other ways (1,4). If a mastoidectomy or resection of any part of the temporal bone is performed the use of thick fasciocutaneous or myocutaneous flaps is mandatory (1,5). 

It is paramount to remember that most of these neoplasms, such as adenoid cystic carcinoma, have a high tendency for recurrence, local infiltration, perineural extension, and metastasis (2,6). An FNAB is an excellent option since it provides adequate diagnosis and sometimes even therapeutic management (2). After complete resection, it is of critical importance to restore function to the patient with facial nerve injury as soon as possible. 

The most important factor that determines the proper success and optimal results in reinnervation procedures is the duration of denervation (7). For immediate reconstruction, if the proximal and distal ends are easily identified, the technique of choice involves either direct repair if possible or a conventional interposition autogenous graft (8). In these cases, some possible options include nerves such as the sural nerve, the great auricular nerve, and the cervical nerve branches (7,9). If the ipsilateral proximal facial nerve end cannot be surgically resettled, alternative donor nerves such as the hypoglossal nerve, masseter nerve and the contralateral facial nerve should be accounted (7,8). 

Vascularized nerve grafts, have shown to result in better nerve regeneration than conventional non-vascularized nerve grafts (9,10) However, there are factors that may affect the degree, speed and regeneration rate regarding the free fasciocutaneous flap such as: a history of previous irradiation in the surgical area, presence of residual scar tissue, poor vascularization of the surgical site due to lack of skin and osseous exposure, more than 60 years of age, and preoperative facial palsy or an altered neurophysiological test (1,8-10). These factors lead to the use of a vascularized nerve graft rather than a conventional nerve graft showing better results. Donor –site morbidity is low and additional surgical time is minimal compared with the time of a single ALT flap transfer (1,4,10). The use of a long non-vascularized nerve graft (more than 6 cm) may be a risk factor for bad nerve regeneration. Vascularized nerve grafts are also used when the nerve gap between the proximal and the distal stumps of the injured facial nerve is more than 6 cm or the graft is performed after reimplantation (8-10). In this case, the patient showed signs of preoperative facial palsy and altered neurophysiological test, presence of residual scar tissue and osseous exposure. Numerous types of vascularized neural grafts have been described such as: the sural nerve graft based on the superficial sural artery or branches of the peroneal artery, lateral femoral cutaneous nerve grafting and its accompanying vessels, deep peroneal nerve grafting and tibial perforating branches, and others (4). Angiographic imaging is recommended prior to grafting. It is important to take into consideration some possible limitations for this technique. Since the nerve graft is attached to the ALT flap, enough length of the nerve graft should be collected (10). The possibility of insufficient nerve branches should also be kept in mind due to anatomical variations among patients. This may require the use of an extra free graft from another site (10). The nerve innervating the vastus lateralis muscle is intimately related to the vascular pedicle of the anterolateral thigh flap, the descending branch of the lateral circumflex femoral artery. This nerve branches extensively as it courses distally through the thigh and usually 3 to 6 nerve branches are found supplying this muscle. These branches are variable in their relation to the vascular pedicle and perforating vessels, and they are usually spared and separated from the vascular pedicle during standard flap harvest (11,12). However, in the present case, the nerve was left attached to vascular pedicle of the flap, and 3 to 4 distal branches to the muscle were dissected and incorporated in the flap. The rest of the dissection of the anterolateral thigh flap was carried out in a standard manner, as described elsewhere. The flap was transferred to the face and vascular end-to-end anastomoses were performed first. The nerve was then used as an interpositional graft, leaving it attached to the vascular pedicle along most of its length. Only the proximal nerve stump and the distal branches were separated from the vascular pedicle, in order to allow a tension-free end to end neurorrhaphy. Finally, the de-epithelialized flap was positioned over the parotidectomy defects and the skin was closed. 

**Fig 3:**
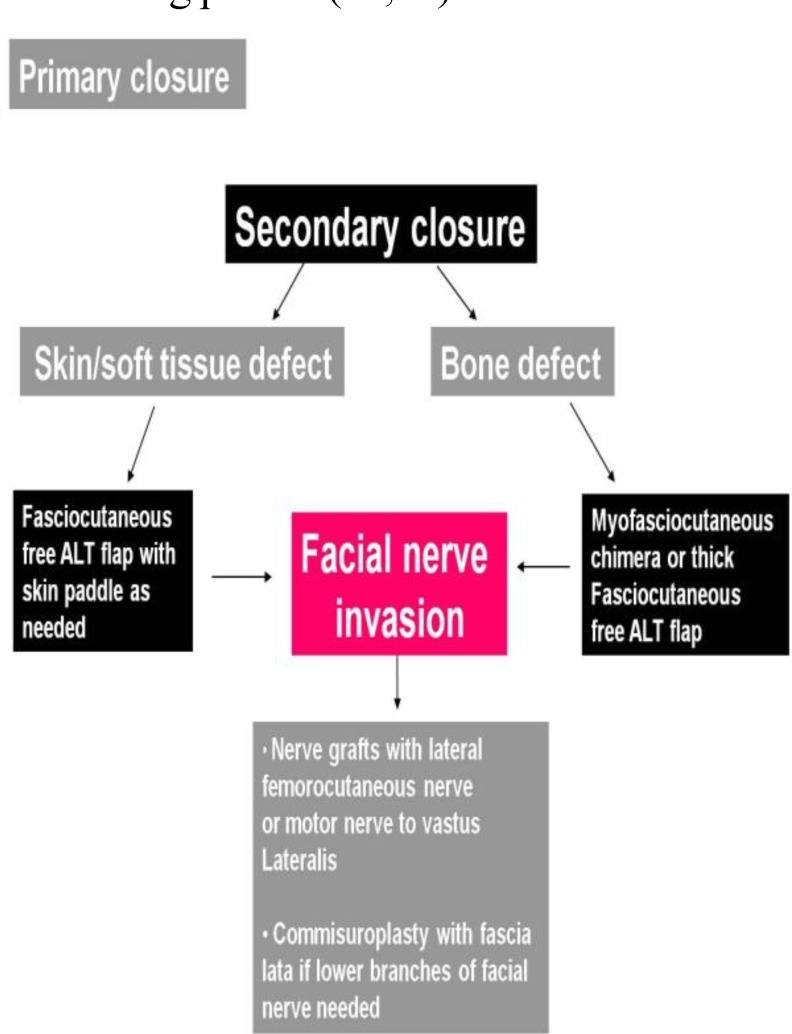
Therapeutic reconstructive algorithm for secondary closure

Depending on the particular defect found, a specific reconstructive surgical strategy should be tailored for each patient (1,8,10) (Fig. 3). The target should be to restore any missing skin and restore the normal facial mass and silhouette with an adequate flap. Potential disadvantages of this approach may include weakness to the vastus lateralis muscle.

However, in our experience most patients recover a normal knee extension after 6 months, as described by other authors. This technique provides a well vascularized nerve, with a favorable length, caliber, and branching pattern (11,12).

## Conclusion

In complex head and neck defects following a total parotidectomy, the extended free fasciocutaneous ALT flap with a vascularized nerve graft is ideally suited for the reconstruction of the injured site. Donor–site morbidity is low and additional surgical time is minimal compared with the time of a single ALT flap transfer (4,10).

Facial retraining therapy can complement and enhance the final results of surgical reanimation techniques (7). Performing this technique grants outstanding functional results and positive aesthetic outcomes; but above all, the patient’s satisfaction and expectations are fulfilled.
